# Pre‐and post‐HSCT use of TKI therapy for fusion‐driven B‐ALL: A case series of five pediatric, adolescent and young adult patients

**DOI:** 10.1002/cnr2.1901

**Published:** 2023-11-07

**Authors:** Savannah S. Shumock, William C. Temple, Amanda Marinoff, Kathryn Aaronson, Erica Southworth, Simayijiang Xirenayi, Alex G. Lee, Stanley G. Leung, E. Alejandro Sweet‐Cordero, Michelle Hermiston, Christine Higham, Elliot Stieglitz

**Affiliations:** ^1^ School of Medicine University of California, San Francisco California USA; ^2^ Department of Pediatrics Benioff Children's Hospitals, University of California, San Francisco California USA

**Keywords:** acute lymphocytic leukemia, chronic myeloid leukemia, hematopoietic stem cell transplant, tyrosine kinase inhibitor

## Abstract

**Background:**

The development of tyrosine kinase inhibitors (TKIs) has significantly improved survival rates among patients with Philadelphia chromosome (Ph+) B cell acute lymphoblastic leukemia (B‐ALL). Ph‐like B‐ALL patients lack the *BCR::ABL1* translocation but share gene expression profiles with Ph+ B‐ALL. The role of TKIs for Ph‐like patients pre‐ and post‐hematopoietic stem cell transplantation (HSCT) is not yet clear.

**Case:**

Here we present five cases of pediatric, adolescent, and young adult patients who presented with Ph‐like B‐ALL or CML in B‐ALL blast phase who were treated with personalized TKI regimens pre‐ and post‐HSCT.

**Conclusion:**

This report describes several novel Ph‐like fusions as well as combinations of TKIs with chemotherapy or immunotherapy not yet reported in the pediatric population. This case series provides real‐world experience highlighting the potential application of pre‐ and post‐HSCT use of TKIs in a subset of patients with targetable fusions.

## 1 INTRODUCTION

Leukemia is the most common malignancy diagnosed in childhood, 75% of which are B‐cell acute lymphoblastic leukemia (B‐ALL).^1^ Chronic myeloid leukemia (CML) accounts for less than 5% of cases in children.^2^ Accelerated or blast phase CML is exceedingly rare in pediatric patients.

The Philadelphia chromosome translocation between chromosomes 9 and 22 produces a BCR::ABL fusion protein with constitutive tyrosine kinase activity that can alter cell signaling pathways leading to dysregulated cellular proliferation.^3^ Prior to the development of tyrosine kinase inhibitors (TKIs), only 20%–30% of patients with Philadelphia chromosome‐positive (Ph+) B‐ALL were cured with chemotherapy alone.^4^

The development of TKIs significantly improved survival rates among these patients. Imatinib was the first approved TKI, which works by blocking the adenosine triphosphate (ATP) binding site of the BCR::ABL protein (Table 1). A Children's Oncology Group (COG) phase II/ trial studied the effects of using chemotherapy with imatinib in pediatric patients with Ph+ B‐ALL and found that the 5‐year event‐free survival (EFS) was 70 ± 12%.^5^ However, in many adult patients imatinib was found to have reduced efficacy due to the acquisition of resistant mutations. This prompted the development of second‐generation TKIs such as dasatinib and nilotinib, which were more potent than imatinib and active against many mutations resistant to imatinib. A COG study using dasatinib with intense chemotherapy for Ph+ ALL resulted in a 5‐year EFS of 60 ± 7% and an overall survival of 86 ± 5%.^6^ As resistance to second‐generation TKIs became apparent, third‐generation TKIs such as ponatinib were developed and demonstrated efficacy against *ABL* mutations including T315I which conferred resistance to first and second‐generation TKIs. Ponatinib was thus approved as a second‐line treatment for CML and Ph+ B‐ALL. Clinical trials have since demonstrated that TKI use in combination with chemotherapy has similar outcomes compared to chemotherapy and TKI followed by HSCT.^5^

**TABLE 1:** Summary of chromosomal fusions found on next‐generation sequencing.

Patient	Chromosome fusion	Pathogenesis of mutation
1	*BCR::ABL1*, *T315I* mutation	Constitutively active tyrosine kinase Activation of RAS‐MAPK pathways
2	*NUP214::ABL1*	Constitutively active tyrosine kinase
3	*RPL11::PDGFRB, CD74::PDGFRB*	Constitutively active tyrosine kinase
4	*EPOR::IGH*	Activation of JAK‐STAT signaling pathways
5	*ETV6::PDGFRB, PRKCA::PDGFRB*	Constitutively active tyrosine kinase

Improved genetic and transcriptomic sequencing has revealed a new subtype of B‐ALL that is similar to Ph+ B‐ALL but lacks the canonical *BCR::ABL1* fusion that defines Ph+ B‐ALL. This entity designated Ph‐like B‐ALL accounts for roughly 15% of B‐ALL cases and increases in prevalence with age.^7^ The alterations involved in Ph‐like B‐ALL lead to over‐activation of several cytokine receptors and kinase signaling pathways, which has raised the possibility of using TKIs in treating this subtype. In particular, Ph‐like B‐ALL patients with ABL class fusions (i.e., *ABL1, ABL2, CSF1R, LYN, PDGFRA, PDGFRB*) have been shown in vitro and in vivo to respond to TKIs including imatinib and dasatinib.^8^, ^9^ Ph‐like B‐ALL patients with overexpression of CRLF2 have also been shown to respond to JAK/STAT inhibitors such as ruxolitinib in vitro and in vivo.^9^ There are currently prospective clinical trials testing these respective strategies including trials NCT03571321, NCT04501614, and NCT02420717.

To date, TKIs have most commonly been used in Ph‐like patients alone, in conjunction with chemotherapy regimens, and/or as a bridge to HSCT in high risk, relapsed, or refractory patients.^7^ Minimal data is available regarding the use of TKIs post‐transplant to prevent relapse. Here we present five cases of pediatric, adolescent, and young adult B‐ALL, including several novel fusions where TKIs were used alone and in combination, pre‐ and post‐HSCT.

## 2 METHODS

Patients aged 0–29 years treated with TKIs for Ph‐like ALL or Ph+ CML in B‐lymphoid blast crisis from January 1, 2017, to December 31, 2022, with clinical follow‐up until June 1, 2023, were eligible for inclusion. Patients who received TKIs on clinical trials were excluded from this study. All patients or their guardians in this case series consented to a tissue bank study which was approved by the Institutional Review Board the University of California, San Francisco (UCSF) Benioff Children's Hospitals and conducted in accordance with the Declaration of Helsinki.

RNA was extracted using standard methods. RNASeq libraries from bone marrow were made using the TruSeq Stranded RNA kit (20 020 594; Illumina), in accordance with the manufacturer's instructions. All manufacturer's controls were used in preparation. The library was analyzed for size, concentration, and presence of primer dimer on the TapeStation 4200 using the HS D1000 assay (Agilent). RNASeq revealed in‐frame fusions using STAR‐fusion and Arriba.^10^, ^11^

## 3 RESULTS

Patient 1 was diagnosed with CML in accelerated phase at age 17 with a detectable *BCR::ABL1* translocation (Table 2). He was started on dasatinib but four months later progressed to Ph+ CML in B‐lymphoid blast crisis. *ABL1* mutation testing was notable for a *T315I* mutation, so he was started on ponatinib in combination with a standard 4‐drug induction. After one month, ponatinib was held due to acute hepatotoxicity and coagulopathy, but once these laboratory values normalized, he was restarted on ponatinib and tolerated therapy well. After one cycle of 4‐drug induction with ponatinib, the patient became flow minimal residual disease (MRD) negative but was still deep sequencing (DS)‐MRD positive (1 copy). Blinatumomab was added to ponatinib for the next cycle, and he became flow MRD and DS‐MRD negative; but remained *BCR::ABL1* positive by reverse transcription polymerase chain reaction (RT‐PCR). He received a haplocompatible αβ T‐cell depleted peripheral blood stem cell transplant (PBSCT) from his sister followed by 6 donor lymphocyte infusions (DLIs) for mixed chimerism and persistent *BCR::ABL1* positivity by RT‐PCR. Three months after his PBSCT, ponatinib was reinitiated as maintenance therapy with the goal of remaining on TKI therapy for two years post‐PBSCT. He was found to have 100% donor chimerism 6 months after transplant. MRD surveillance was performed monthly for the first 3 months after transplant, bimonthly for the next 6 months, then every 3 months for the next year. He remains negative by flow MRD, DS‐MRD and RT‐PCR for *BCR::ABL1*, 22 months post‐transplant (Figure 2).

**TABLE 2:** Summary of treatments and outcomes.

Patient	Diagnosis	Fusion	MRD with flow cytometry prior to TKI	DS‐MRD prior to TKI (# cells/million)	Treatment with TKI	MRD with flow cytometry post‐TKI‐ time post‐TKI	DS‐MRD post‐TKI (# cells/million)‐time post‐TKI	RT‐PCR results
1	CML that progressed to Ph+ CML with B‐lymphoid blast phase	*BCR‐ABL1*, *T315I* mutation	2% 94.0% 0% 0%	N/A 1 0 0	Pre‐HSCT: First: dasatinib Second regimen: 4‐drug induction Third regimen: blinatumomab and ponatinib for 1 cycle Post‐HSCT: ponatinib	0% – 1 month 0% – 1 month	0 – 1 month 0 – 1 month	106.650 + − 1 month 0 – 1 month
2	B‐ALL	*NUP214::ABL1*	31.6% 0% 0%	391 170 3 0	Pre‐HSCT: First: High‐dose methotrexate, vincristine, and dasatinib Second regimen: blinatumomab and nilotinib Post‐HSCT: nilotinib	0% – 1 month 0% – 1 month	0 – 1 month 0 – <1 – 1 month	N/A
3	Ph‐like B‐ALL	*CD74::PDGFRB; RPL11::PDGFRB*	0%	0 – <1	Post‐HSCT: dasatinib	0% – 1 month	0– 1 month	N/A
4	Ph‐like B‐ALL	*EPOR::IGH*	0%	0	Post‐HSCT: ponatinib Post‐HSCT: inotuzumab	0% – 1 month 23.7% – 3 months 0% – 1 month	0 – 1 month 61 016 –3 months 3 – 1 month	N/A
5	Ph‐like B‐ALL	*PRKCA‐PDGFRB*	15% 0% 1.5% 0%	N/A 0 113 0	Pre‐HSCT: consolidation therapy per AALL1131 with dasatinib Second regimen: dasatinib and blinatumomab Post‐first‐HSCT: First: dasatinib Second regimen: etoposide, cyclophosphamide, and dasatinib Post‐second‐HSCT: dasatinib Post‐ inotuzumab	5.8% – 0.0% – 1 month 1.0% – 12 months 1.5% – 1 month 0% – 1 month 0% – 16 months 0% – 1 month	N/A 0 – 1 month 3 – 12 months N/A 0 – 1 month 29 – 16 months 0 – 1 month	N/A

Patient 2 was diagnosed with B‐ALL at age 21 and treated by adult hemato‐oncologists with a modified hyperfractionated cyclophosphamide, vincristine, doxorubicin, and dexamethasone (Hyper‐CVAD) regimen. His end of induction (EOI) flow MRD was positive (1.3%). He proceeded to receive methotrexate, cytarabine, and rituximab, followed by 3 cycles of inotuzumab but remained flow MRD positive (from 3.8% to 0.5%). After an overt relapse, he received one cycle of blinatumomab with no response followed by 2 cycles of clofarabine, cyclophosphamide, and etoposide but remained flow MRD positive (1.1%). He was then referred to our tertiary‐care pediatric hospital for evaluation to receive chimeric antigen receptor‐T (CAR‐T) cells. He underwent leukapheresis and received a standard 4‐drug reinduction for one cycle while awaiting CD19 CAR‐T cell manufacturing. The patient remained with measurable disease (31.6%) after one cycle. During this time, clinical‐grade next‐generation sequencing identified a *NUP214::ABL1* fusion (Table 2), so the patient was treated with a salvage regimen of interim maintenance‐like ALL therapy with high‐dose methotrexate (HDMTX), vincristine, and dasatinib. He later developed grade 2 pulmonary and gastrointestinal toxicity so dasatinib was discontinued due to concern for drug‐related toxicity. He was then diagnosed with an invasive pulmonary fungal infection, so to reduce the risk of further myelosuppression, cytotoxic chemotherapy was stopped, and the patient was started on a cycle of blinatumomab in combination with nilotinib. After this salvage regimen, he became flow MRD and DS‐MRD negative. He proceeded to receive a 12/12 matched sibling allogeneic PBSCT and did not have any significant peri‐HSCT complications. Three months after HSCT, nilotinib was resumed and TKI therapy was continued for two years post‐PBSCT. MRD surveillance was performed bimonthly for the first 6 months after transplant, then every 3 months for the next 6 months, and then annually. He remains negative by flow MRD and DS‐MRD two years post‐HSCT (Figure 2).

**FIGURE 1:**
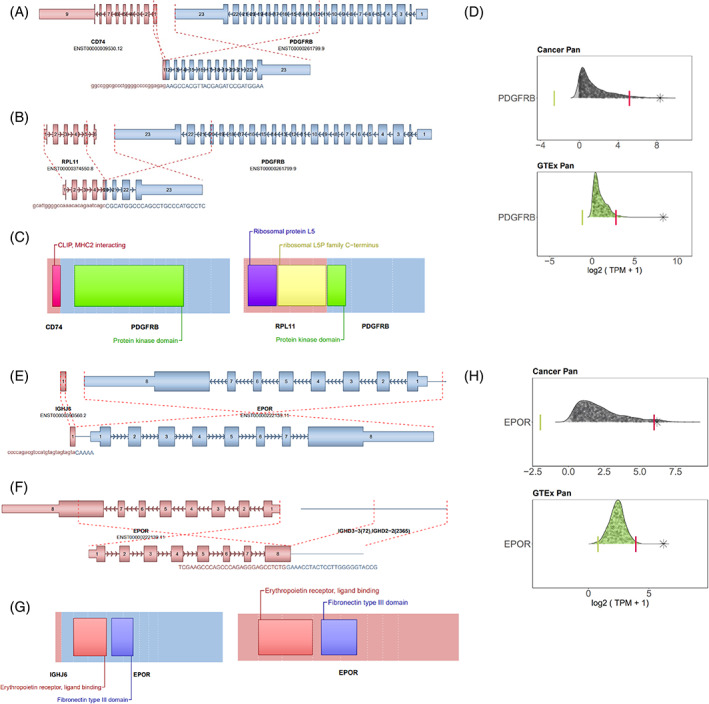
RNASeq reveals the *PDGFRB* and *EPOR* fusions. (A,B) Illustration of *PDGFRB* fusions. The top rows display the gene structure by exons, while the bottom rows show the final fusion predictions. (C) predicted domains retained as a result of the fusions, *CD74* and *RPL11* respectively. (D) Tukey outlier analysis. The top represents the expression comparison of the sample and leukemia diagnosis samples (*N* = 1627). Each dot represents a sample's expression, while the bottom section compares the sample to normal whole blood and lymphocyte samples (*N* = 444). The asterisk indicates the sample in question. Green and red vertical lines mark the outlier borders (below Q1 – 1.5IQR or above Q3 + 1.5IQR). (E,F) Representation of the *EPOR* fusion. (G) The predicted fusion domains of the resulting gene fusions. (H) Tukey outlier analysis for the EPOR gene.

**FIGURE 2:**
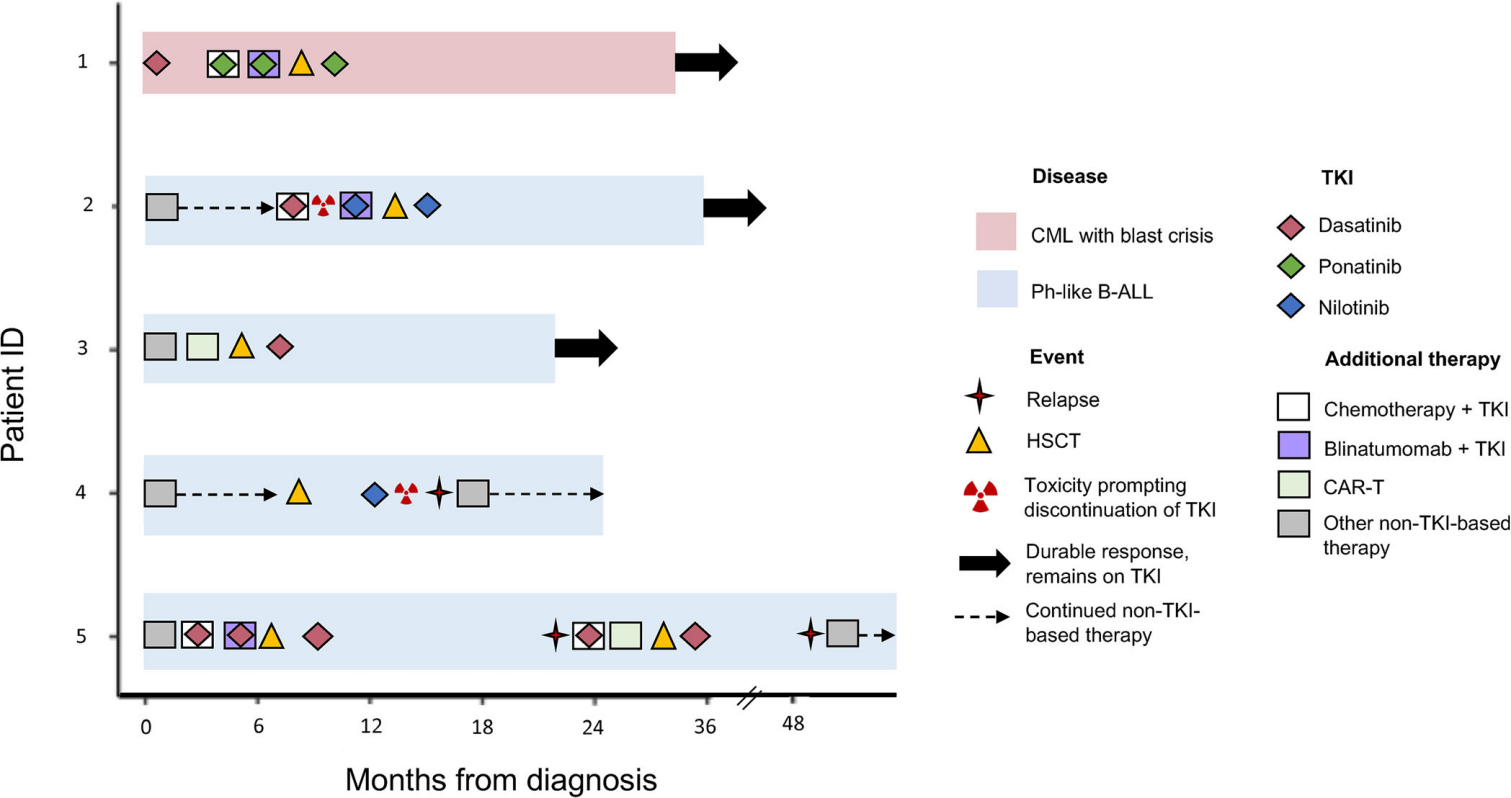
Swimmer plot of patients' clinical courses. Swimmer plot showing the clinical course of each patient over time. Each bar represents one patient, color‐coded based on diagnosis (CML with blast crisis, pink; Ph‐like B‐ALL, blue). Tyrosine kinase inhibitors (TKI) are depicted by the diamonds and color‐coded by type (dasatinib, red; ponatinib, green; nilotinib, blue). Additional therapy is shown by the colored squares (white, chemotherapy in combination with TKI; purple, blinatumomab in combination with TKI; green, CAR‐T cell infusion, gray, other non‐TKI‐based therapy). Overlapping diamond and square indicates combination therapy. Dates of relapse (four‐pointed star), hematopoietic stem cell transplantation (HSCT, yellow triangle), toxicity prompting discontinuation of TKI (hazard symbol), durable response while patient remained on TKI (thick black arrows) and continued non‐TKI‐based therapy (dashed arrows) are depicted by additional symbols.

Patient 3 was diagnosed at age 7 with high‐risk Ph‐like B‐ALL with *CD74::PDGFRB* (out of frame) and *RPL11::PDGFRB* (in frame) fusions (Table 2, Figure 1A,B). He received a standard 4‐drug induction. His EOI flow MRD was positive (82.2%), so he underwent apheresis for CD19 CAR‐T cells and received bridging therapy with cyclophosphamide and etoposide. After CAR‐T infusion he was flow MRD negative but remained DS‐MRD positive (7 copies/million). Two months after the CAR‐T cell infusion he received an αβ T‐cell depleted haplocompatible PBSCT from his father followed by 6 DLIs for persistent mixed CD3 chimerism. Two months after his HSCT he was started on dasatinib with the goal of remaining on TKI therapy for two years post‐PBSCT. MRD surveillance was performed monthly for the first 3 months after transplant, bimonthly for the next 6 months, then every 3 months for the next 9 months. He remains on dasatinib and in CR at 18 months by both flow and DS‐MRD (Figure 2).

Patient 4 was diagnosed with Ph‐like B‐ALL at age 14 presenting with an *EPOR::IGH* fusion (Table 2, Figure 1C,D). She was treated with a standard 4‐drug induction. Her EOI flow MRD was positive (2.3%) and end of consolidation (EOC) flow MRD was positive (0.09%). She then received 3 infusions of HDMTX, in addition to vincristine, cytarabine, and 6‐mercaptopurine while awaiting CAR‐T cell infusion. She received CD19 CAR‐T cells, but lost B‐cell aplasia by day 41. She then received an additional CAR‐T cell infusion from the same batch but had disease progression eight days later with 74% CD19+ circulating blasts in the peripheral blood. She began reinduction with inotuzumab monotherapy and became flow MRD negative but remained DS‐MRD positive (4 copies/million) after 1 cycle. She then underwent an T‐cell depleted haploidentical PBSCT from her mother. Her +27 post‐HSCT marrow was negative by flow MRD and DS‐MRD. She was started on ponatinib on day +48 after HSCT but developed transaminitis and myelosuppression and was therefore taken off ponatinib after 3 weeks and relapsed on day +89. She was next treated with ruxolitinib in combination with vincristine, dexamethasone and asparaginase but had no response to therapy (M3 marrow). She was then treated with single agent inotuzumab for a second time and became flow MRD negative after 1 cycle but DS‐MRD positive (3 copies/million). This patient is day +10 following a second allogeneic HSCT (Figure 2).

Patient 5 was diagnosed at age 3 with high‐risk Ph‐like B‐ALL with a complex rearrangement involving *ETV6::PDGFRB* and *PRKCA::PDGFRB* fusions (Table 2). He received a standard 4‐drug induction. His EOI flow MRD was positive (9%) and proceeded to consolidation therapy as per AALL1131 with the addition of dasatinib. He remained EOC flow MRD positive (1.9%). He received interim maintenance with HDMTX but remained flow MRD positive. He next received 1 cycle of blinatumamab and dasatinib and became flow MRD and DS‐MRD negative. He received a 12/12 matched sibling PBSCT followed by 6 DLIs for mixed CD3 chimerism. One‐month post‐HSCT, he was negative by flow MRD and DS‐MRD and was then started on dasatinib as maintenance therapy. He was found to be 100% donor chimerism five months after transplantation. Approximately one year later he presented with rising DS‐MRD positivity and subsequent flow MRD positivity (1%). He started on etoposide, cyclophosphamide, and dasatinib as a bridge to infusion with bispecific CD19/22 CAR‐T cells and remained flow MRD positive (1.5%) prior to the CAR‐T infusion. One month after CAR‐T infusion, the patient was negative by flow MRD and DS‐MRD. Two months after his CAR‐T cell infusion he underwent an αβ T‐cell depleted haploidentical PBSCT followed by 6 DLIs for mixed CD3 chimerism. He was then restarted on dasatinib but relapsed 14 months later. He underwent leukapheresis and was treated with single agent inotuzumab as bridging therapy which resulted in nearly 5 months of flow MRD and DS‐MRD negativity. The patient is currently day +8 after an infusion of CD19 CAR‐T cells (Figure 2).

## 4 DISCUSSION

While TKIs are standard of care for patients with CML and Ph+ B‐ALL, there remains a paucity of data in the pediatric setting using TKI therapy in the pre‐ and post‐HSCT settings in patients with Ph‐like B‐ALL. The five patients presented here were treated with TKIs either as monotherapy or in combination with chemotherapy pre‐HSCT and as monotherapy post‐HSCT to help induce and maintain remission, respectively.

Patient 1 was initially diagnosed with CML, progressed to B‐ALL blast phase, and was found to have a *T315I* mutation. The patient was treated with a pediatric‐inspired 4 drug induction with the addition of ponatinib. T315I mutations alter the properties of the ATP binding region on the BCR::ABL protein conferring resistance to the first and second‐generation TKIs through increased steric hinderance and its lack of a threonine residue preventing hydrogen bonding of these drugs with the protein.^12^ Ponatinib was designed to have increased binding affinity, lipophilicity, and oral bioavailability when compared against first and second generation TKIs. The triple bond ethynyl linker of ponatinib allows it to overcome the steric hinderance of the T315I mutation and tightly bind with the kinase active site of BCR::ABL, inhibiting its leukemic stimulation.^12^ An international working group has established expert recommendations for the treatment of pediatric CML.^13^ However, minimal literature exists regarding the benefits of using TKIs in addition to intensive chemotherapy regimens including asparaginase to treat CML in blast phase, particularly in pediatric patients. However, more robust adult data is available, including a retrospective study that compared survival outcomes for 104 adult patients with CML in blast phase treated with intensive chemotherapy in addition to a TKI (*n* = 20) versus a TKI alone (*n* = 56). Compared to being treated with a TKI alone, combination chemotherapy with a TKI resulted in higher rates of CR with incomplete count recovery (33.9% vs. 57.5%, *p* < 0.05) and more patients proceeding to allogeneic stem cell transplant.^14^ Additionally, a single case report of pre‐transplant therapy with ponatinib in an adolescent with CML in blast phase with a *T315I* mutation significantly decreased peripheral blast count and was able to provide a bridge to HSCT. The patient also received ponatinib post‐HSCT due to a molecular relapse, which resulted in a molecular remission.^15^

Patient 2 initially presented with B‐ALL that was unresponsive to multiple therapeutic regimens. After identification of a rare *NUP214::ABL1* fusion, the patient finally achieved remission with the use of nilotinib in combination with other therapies. NUP214 is a component of the nuclear pore complex and mediates nucleocytoplasmic transport, while also regulating the cell cycle.^16^ The *NUP214::ABL1* fusion is a constitutively active tyrosine kinase that is rare among B‐ALL patients. In vitro studies demonstrated that this fusion is sensitive to imatinib and dasatinib, but clinical data is limited.^17^ One report of an adolescent with B‐ALL and a *NUP214::ABL1* fusion found that dasatinib used in combination with chemotherapy was more effective at reducing tumor burden than the same chemotherapy used alone.^18^ Dasatinib is a type 1 inhibitor designed to inhibit ABL and SRC. Type II inhibitors like nilotinib actively compete with ATP binding and have stricter binding requirements. Nilotinib exerts its effect by binding to the ABL1 kinase in its inactive form.^9^ Phase II trials of both dasatinib and nilotinib use in pediatric patients found the safety profiles to be similar to those of adult patients treated with these TKIs.^19^, ^20^ Patient 2 had primary refractory B‐ALL, and only achieved DS‐MRD negativity after receiving nilotinib. This case highlights the potential therapeutic role of TKI therapy *for NUP214::ABL1* fusion positive B‐ALL, a highly refractory subtype of B‐ALL.

Patient 3 was diagnosed with Ph‐like B‐ALL. UCSF500, a DNA‐based next generation sequencing (NGS) panel identified a *CD74::PDGFRB* fusion. Research‐grade RNAseq revealed the *CD74::PDGFRB* fusion, along with four other *PDGFRB* fusions, including *EEF1A1::PDGFRB* and *RPL11::PDGFRB*. The *CD74::PDGFRB* fusion is out‐of‐frame but retains the entire kinase domain. In contrast, *EEF1A1::PDGFRB* and *RPL11::PDGFRB* are both in‐frame. *EEF1A1::PDGFRB* is likely non‐functional since only the last exon remains, while *RPL11::PDGFRB* is in‐frame retaining part of the kinase domain and is most likely the main driver (Figure 1A–C). *PDGFRB* was also upregulated on RNASeq (Figure 1D), and the decision was made to initiate dasatinib post‐HSCT for maintenance therapy. CD74 is part of major histocompatibility complex (MHC) class II and is also a receptor that binds macrophage migrating inhibitory factor, regulating transcription and cell survival.^21^ This report describes the use of dasatinib for post‐HSCT maintenance therapy for refractory Ph‐like ALL driven by a *PDGFRB* fusion. This suggests that this strategy may be viable in other leukemias driven by novel fusions with susceptibility to ABL1 kinase inhibitors.

Patient 4 originally presented with an *EPOR::IGH* fusion detected on Foundation Medicine testing and confirmed via fluorescence in situ hybridization that was refractory to multiple rounds of chemotherapy and CAR‐T cell treatments. Research‐grade RNAseq analysis verified the presence of both *EPOR::IGH* and its reciprocal *IGH::EPOR* fusion. As illustrated in Figure 1, the *EPOR::IGH* fusion is likely to result in a truncated yet active *EPOR*, preserving the majority of its functional domain. In the *IGH::EPOR* fusion, a small segment of the *IGHJ6* sublocus is joined upstream of a complete *EPOR* gene, which likely leads to a promoter exchange and a change in its expression. Notably, an outlier analysis reveals that *EPOR* is overexpressed in this particular sample. There are few patients with this gene fusion in the literature who were treated with ponatinib post‐HSCT.^22^ *EPOR* rearrangements have one of the worst outcomes for patients with Ph‐like ALL and represent about 4% of Ph‐like B‐ALL cases.^23^ These gene rearrangements lead to hypersensitivity to erythropoietin stimulation and constitutive activation of JAK–STAT signaling pathways.^21^ EPOR::IGH fusion treatment regimens commonly involve the use of ruxolitinib, a JAK1 kinase inhibitor. While current clinical trials are still assessing the efficacy of ruxolitinib, initial data suggests it may hinder desired post‐HSCT graft‐versus‐leukemia effects, which is why this patient was treated with ponatinib.^24^ Ponatinib was first developed as a CML therapy that retains activity against mutations that yield resistance to first and second generation TKIs. Little data is available regarding the use of ponatinib in pediatric patients. A retrospective cohort study of 11 children with CML and 3 children with Ph+ ALL, found that ponatinib used alone or in combination with chemotherapy produced impressive molecular responses in 50% of cases, and no vascular complications were observed.^25^ Patient 4 had difficulty tolerating ponatinib post‐HSCT and eventually relapsed. It is possible that limited exposure to ponatinib contributed to her subsequent relapse. She was also treated with ruxolitinib in combination with a 3‐drug reinduction, which was not effective. Further studies are necessary for patients with *EPOR::IGH* fusions to identify the optimal use of TKIs both pre‐ and post‐transplant.

Patient 5 presented with Ph‐like B‐ALL with a rare *PRKCA::PDGFRB* fusion as part of a complex rearrangement involving *ETV6::PDGFRB*. The patient was treated with dasatinib before and after both HSCTs but relapsed both times. PRKCA is part of the protein kinase C family and phosphorylates several signaling molecules involved in pathways for cell proliferation, apoptosis, and differentiation including ERK–MAPK, NF‐κB and PI3K/AKT pathways. Overactivation of PRKCA has been associated with tumorigenesis in malignancies including lung cancer, breast cancer, colon carcinomas, and other hematologic malignancies.^26^ Ph‐like ALL often involves fusions in *PDGFRB* which induces expression of cyclin D1 and produces tyrosine kinase activating fusions.^20^ Presently there are over 20 fusions reported with *PDGFRB*, and case reports have shown promising results when treated with first‐ and second‐ generation TKIs. An in vitro study in murine pre‐B cell lines analyzing the effect of TKIs on various Ph‐like fusion proteins found that in vitro PDGFRB fusions were sensitive to dasatinib.^27^ In a pediatric patient with refractory ALL with an *ATF7IP::PDGFRB* fusion, complete remission was achieved with dasatinib treatment.^28^ A retrospective cohort study conducted by COG looked at 1389 patients diagnosed with high‐risk B‐ALL and found that Ph‐like ABL‐class fusions including *PDGFRB* were sensitive to dasatinib and could be added to backbone chemotherapy in such cases.^29^

In this report, we provide real‐world evidence that the use of TKIs targeting fusions in combination with chemotherapy and immunotherapy may be a promising strategy for the treatment of Ph‐like B‐ALL across a range of driver fusions and in one case of CML with blast crisis. We demonstrate several exceptional responders, including in cases of leukemias refractory to multiple lines of prior therapy. While two of the five patients presented had to stop TKI therapy due to adverse outcomes, these patients recovered promptly and were switched to alternative TKIs. Additionally, integrated genetic profiling provided opportunities to incorporate targeted therapies into treatment regimens of Ph‐like B‐ALL, and we have demonstrated clinical responses using this approach, compared to standard chemotherapy alone, in a subset of this case series. As such, diagnostic screening tools that can identify targetable driver alterations should be incorporated upfront at diagnosis. Limitations to this report include the small sample size, that several patients were started on TKIs in combination with immunotherapy pre‐HSCT, and that several patients were started on TKI therapy post‐HSCT while already in remission, limiting our ability to draw conclusions about the specific contribution of TKI therapy. In the future, MRD monitoring of the fusion transcripts by RT‐PCR should be performed pre‐ and post‐HSCT to assess response to TKI use. However, this case series contributes to reports of safely combining TKIs into personalized treatment regimens for Ph‐like and CML in blast phase B‐ALL patients at high risk of relapse.

## AUTHOR CONTRIBUTIONS

**Savannah Shumock:** Conceptualization (equal); investigation (equal); writing – original draft (lead); writing – review and editing (lead). **William C Temple:** Investigation (equal); writing – review and editing (equal). **Amanda Marinoff:** Investigation (equal); writing – review and editing (equal). **Kathryn Aaronson:** Investigation (equal). **Erica Southworth:** Investigation (equal). **Simayijiang Xirenayi:** Investigation (equal). **Alex G Lee:** Investigation (equal); methodology (equal). **Stanley G Leung:** Investigation (equal); methodology (equal). **E. Alejandro Sweet‐Cordero:** Investigation (equal); methodology (equal). **Michelle Hermiston:** Conceptualization (equal); investigation (equal); writing – review and editing (equal). **Christine Higham:** Investigation (equal). **Elliott Stieglitz:** Conceptualization (lead); investigation (equal); methodology (equal); resources (equal); writing – review and editing (equal).

## CONFLICT OF INTEREST STATEMENT

The authors have explicitly stated that there are no conflicts of interest in connection with this article.

## ETHICS STATEMENT

The study was conducted at the University of California, San Francisco Benioff Children's Hospitals in accordance with the Declaration of Helsinki.

## PATIENT CONSENT STATEMENT

All patients have consented to the usage of their clinical vignettes and data.

## Data Availability

The data that support the findings of this study are available from the corresponding author upon reasonable request.
